# Case Report: What—or who—killed Frank Ramsey? Some reflections on cause of death and the nature of medical reasoning

**DOI:** 10.12688/wellcomeopenres.17759.1

**Published:** 2022-05-23

**Authors:** Cheryl Misak, C David Naylor, Mark Tonelli, Trisha Greenhalgh, Graham Foster

**Affiliations:** 1 University of Toronto, Toronto, Canada; 2 University of Washington, Seattle, Seattle, USA; 3 University of Oxford, Oxford, UK; 4 Queen Mary, University of London, London, UK

**Keywords:** Weil's disease, gallstones, primary sclerosing cholangitis, uncertainty, Frank Ramsey

## Abstract

Philosopher Frank Ramsey died in 1930 aged only 26. There has been much speculation about the nature of his final illness and the sequence of events which led to his death. To prepare this case report, we traced Ramsey’s medical records and combined them with an extensive and unique dataset of contemporaneous sources. We use these to evaluate three possible explanations for Ramsey’s illness and its unexpectedly fatal trajectory—infectious (Weil’s disease), autoimmune (primary sclerosing cholangitis) and obstructive (gallstones). We explore how uncertainty surrounding each of these possibilities might have influenced Ramsey’s doctors’ thoughts and actions, including their ill-fated decision to perform the emergency operation that appears to have precipitated his final decline.  We then reflect on the unfinished opus on which Ramsey was working when he died—on the nature of truth and how humans reason under conditions of uncertainty. We end with some thoughts linking Ramsey’s death to his philosophy.

## Consent

For this historical case, written informed consent for publication of the patient’s clinical details and clinical images was obtained from the surviving grandson of the patient and also from the Caldicott Guardian and Information Governance Manager of Guys and St Thomas’s NHS Foundation Trust.

## Introduction

Frank Ramsey, a brilliant polymath and Fellow of King’s College, Cambridge, died on January 19, 1930 in Guy’s Hospital, London, at the age of 26. By that time, he had already made extraordinary contributions to three academic disciplines—mathematics (founding an important branch of combinatoric mathematics now called Ramsey Theory), economics (founding optimal utility theory, and publishing classic papers on optimal taxation and savings), and philosophy (while still a teenager, he translated Ludwig Wittgenstein’s first book and published a far-reaching critique of it and he then made important contributions in the philosophy of language, the philosophy of science, and truth theory). As one Bloomsbury writer (Lytton Strachey) wrote to another (Dadie Rylands): “The loss to your generation is agonizing to think of – and the world will never know what has happened – what a light has gone out. I always thought there was something of Newton about him – the ease and majesty of the thought – the gentleness of the temperament” ( Misak, 2020: 421).

Whilst it is known that Ramsey died after an acute illness characterized by several weeks of jaundice, the exact cause of his death remains a mystery. In this paper, we try to piece together Ramsey’s final illness trajectory, speculating on the differential diagnosis and the proximal cause of death, using biographical archival material and recently unearthed (though tantalizingly incomplete) hospital case notes. Our research question was: “what—or who—killed Frank Ramsey, and what can we learn from studying his final illness about medical decision-making then and now?”.

This exercise in itself may shed some useful light on modes of medical reasoning but also illustrates the enduring relevance of Ramsey’s thought to disciplines such as medicine. At the time of his death, Ramsey was working on a book about how we reason under conditions of uncertainty—an unfinished magnum opus which was drawing together interdisciplinary insights from philosophy, psychology, economics, and mathematics. Ramsey’s thoughts on scientific hypotheses, subjective probabilities, and the nature of truth resonated for the authors as we grappled with provisional initial diagnoses and competing explanations for the ultimate cause of Ramsey’s death. We end with some thoughts linking the philosopher’s death to his philosophy.

## Approach

We sought to undertake an interdisciplinary analysis, informed by clinical, historical and philosophical insights, of Ramsey’s final illness. This aimed to build on preliminary discussions held among three of the authors (CM, MT and DN) in 2019. They, along with TG and GF, represented a range of sub-disciplines and had come together through their academic networks. We collaborated over a period of four months (July–October 2021) by email and video conference to develop and enrich an account of events and possible underlying explanations. GM, a professor of liver medicine, presented Ramsey’s ‘case’ in a weekly multidisciplinary team meeting of liver clinicians. Provisional diagnoses were explored both through targeted searches of the contemporary literature and also using medical articles and books that had been available in 1930, so as to gauge how the clinicians of the day might have reasoned. As described below, we also consulted specialists in one rare disease.

The following sources had previously been collected by CM as part of a wider dataset to inform a book-length biography of Ramsey ( Misak, 2020):

- Letters from Ramsey’s wife Lettice Ramsey to Ludwig Wittgenstein, letters from Ramsey to his wife, and other archival materials
- Conversation with Lionel Penrose’s son Roger, 2019

To this collection, TG sought and found the surviving elements of Ramsey’s medical records from Guy’s Hospital, London, which were sourced from London Metropolitan Archives in July 2021 using their professional searching service (see acknowledgements). Archivists looked for, but did not find, a coroner’s report.

Whilst the original aim of the study was to pin down the cause of death, it became increasingly evident that each candidate diagnosis had some arguments in favour but also some arguments against. The study thus evolved into a contemplation on the nature of uncertainty and the sometimes fruitless search for truth. Given that Ramsey himself had written on this very topic, we went on to consider how his own (unfinished) writings might provide relevant insights.

The patient himself is, of course, long deceased, but we contacted surviving members of Ramsey’s family, notably his grandson Stephen Burch, and received his permission to undertake the study and to reproduce a chart from the case notes.

## Case history

In this section, we summarise what is known of the case. Ramsey was transferred from his home in Cambridge to Guy’s Hospital on 10 January 1930 for investigation and management of worsening jaundice. His medical history was essentially unremarkable. He had neonatal jaundice and ill-defined “digestive” issues as a child. But by adolescence, he was a competent athlete and as an adult appeared to enjoy good health. He was a tall man tending towards weight gain—in his early 20’s, he weighed nearly 17 stone: 240 lbs/108 kilos. Assuming his height was around 6 feet 4 (1.93 metres), his body mass index was about 29: overweight but not obese. He was physically active, including vigorous hiking and wild swimming. He was described as having “tremendous vitality”. He was a member of the Bloomsbury set—a group of intellectuals and artists who lived life to the full and were known to have open marriages, as did Ramsey and his wife, the former Lettice Cautley Baker, who became a renowned photographer. While Lettice had multiple sexual partners, Ramsey appears to have had only one other: Elizabeth Denby, a senior civil servant and social housing reformer.

 In mid-November 1929 Ramsey developed an acute febrile illness after a College feast (a formal occasion involving many courses of rich food, washed down with much alcohol). Nobody else attending the feast is known to have become ill, and there seem to have been no sick contacts, though Lettice developed a brief febrile illness in late November from which she recovered uneventfully. Within 10 days of the onset of fever, Ramsey developed jaundice with malaise, but no abdominal pain and no signs of bleeding or bruising. Progressive jaundice and malaise persisted for over six weeks. His cognitive status remained more or less normal throughout this period (no suggestion of encephalopathy), though he sent notes apologising to academic colleagues that he was unable to focus on his work.

On January 10, 1930 Lettice contacted her uncle, Robert Davies-Colley, a senior surgeon at Guy’s Hospital, who arranged for Ramsey's admission. He was evaluated by a physician and a surgeon who apparently concurred on proceeding with a laparotomy to evaluate for biliary stones or other treatable cause of obstruction. Lettice wrote to Wittgenstein with an account of her understanding on Wednesday, January 15:

Frank was operated on Saturday afternoon, because, after 8 weeks in bed he showed no improvement. My uncle, who’s a surgeon, came from London to see him and thought that he should be brought to London to Guy’s hospital. So we took an ambulance & came. Then there was a consultation with a physician (as opposed to a surgeon) & firstly they agreed that an op would be best. They found his gall bladder very inflamed & are draining it. There was no stone. I have seen Frank twice today, for a few moments. He is still too much under the effect of drugs to like any company . . . The dose of morphia that is always given after an op had a bad effect on him & he had to have something to counteract it. ( Misak, 2020: 418)

The above quote contains what appears to be an error by Lettice, since the actual date of the operation was Tuesday 14 ^th^; Ramsey died on Sunday 19 ^th^. While the surgery did not reveal an obstruction, “the whole liver and kidneys were found in a frightful condition”. ( Misak, 2020: 425)

The records suggest that a partial autopsy ("P.M. Examination of the abdominal organs") was performed but no findings from this are available. The death certificate simply read:

      Cause of Death:      1 (a) Cholangitis (b) Infection of smaller bile ducts

                                      2 Hepatitis.

                                                      P.M. Examination of abdominal organs

Lettice said that Frank “died of an infected liver”. Davies-Colley told Ramsey’s father it was “a degenerative disease of the liver which was bound to end fatally”. Lionel Penrose (father of British genetics and himself a medical doctor) was one of Ramsey’s best friends, and apparently had a different view. He would later allude to the possibility that medical malpractice was the cause of death and he was wracked with guilt about not having been around to step in and save his friend ( Misak, 2020: 425).

## Differential diagnosis: Leptospirosis? Primary Sclerosing Cholangitis? Surgical mishap?

In 2019, before the hospital case notes were unearthed, MT and DN (a medical intensivist and an internist, respectively) were helping CM (a philosopher) think through Ramsey’s cause of death for her biography. MT made a surmise, based on the biographical materials and death certificate: Leptospirosis, particularly the severe form, Weil’s disease, caught from swimming in the River Cam. MT’s reasoning started with the observations that Ramsey had no antecedent medical problems to suggest a chronic illness and never developed encephalopathy (ruling out some common causes of acute liver failure). Supporting this speculation, the course of disease broadly fit the classical description of leptospirosis, a febrile illness followed by jaundice that typically involves both the liver and the kidneys. Leptospirochetes have been isolated from samples taken from the Cam in recent years. The physicians and surgeons caring for Ramsey did not discover a firm alternative diagnosis despite a laparotomy and post-mortem examination. DN was sceptical, as he thought that the incubation period was such that Ramsey would have had to be swimming in the river in late October—a bit cold and hence unlikely. But it turned out that the weather in October 1929 was headline-worthy warm. DN concluded that the leptospirosis hypothesis was not unreasonable. CM’s biography floated the surmise, saying also that the surgical procedure, when Ramsey was already very unwell and jaundiced, “undoubtedly hastened his death”. ( Misak, 2020: 425)

TG had trained as a general practitioner and been taught the maxim that rare symptom combinations are more often caused by atypical presentations of common diseases than by rare diseases. She suggested in the spring of 2021 that we take a new look at Ramsey’s cause of death, hypothesising that it might be explained by an unusual complication of acute viral hepatitis (which was very common at the time), rather than by leptospirosis (which was rare). We invited the opinion of a hepatologist (GF, a professor of liver medicine who edits the journal *Viral Hepatitis*). GF was even more sceptical about the leptospirosis hypothesis, as he felt the disease course was too slow, and the lack of encephalopathy unusual, as it meant that Ramsey did not have the acute liver failure that characterizes the fatal form of leptospirosis known as Weil’s disease.

Following discussion among his multidisciplinary team, GF posited the autoimmune disorder primary sclerosing cholangitis. Supporting this diagnosis, the condition is commoner in men than women, can present in early adulthood, is caused by an inflammation and strictures of the bile ducts, and causes portal hypertension—a complication which carries a very high mortality if operated on. In this scenario, Ramsey’s febrile illness following the College feast (perhaps suggesting an infectious process) was perhaps a red herring. Rather, the picture of a gradually worsening jaundice over several weeks followed by rapid deterioration (including tachycardia and severe anaemia requiring transfusion) after a laparotomy and death soon after pointed more towards a non-infectious hepatic disorder that was made fatal by surgical intervention. This, however, does not fully explain why Ramsey’s kidneys, viewed per-operatively, as well as his liver, would have been in what his wife called “a frightful condition”. Nor does it fit well with the reports of Ramsey’s robust health and absence of typical symptoms prior to his acute illness, as primary sclerosing cholangitis generally has a more indolent course with prior episodes of obstructive jaundice.

In short, the narrative history accommodated multiple potential diagnoses, though none without having to acknowledge some inconsistencies.

## Clinical information: Elucidating? Confounding?

Archivists at Guy’s hospital supplied us with a photocopy of the surviving parts of Ramsey’s hospital record, providing further (though incomplete) details of the case. The Case Notes (completed by the Registrar of Deaths), which were not made available to CM during the writing of her biography, include the following entry:

Nature of Case: Inflammation. Chronic spirochaetae. Liver. Hepatitis
Result: Laparotomy-Death

**Figure 1.**
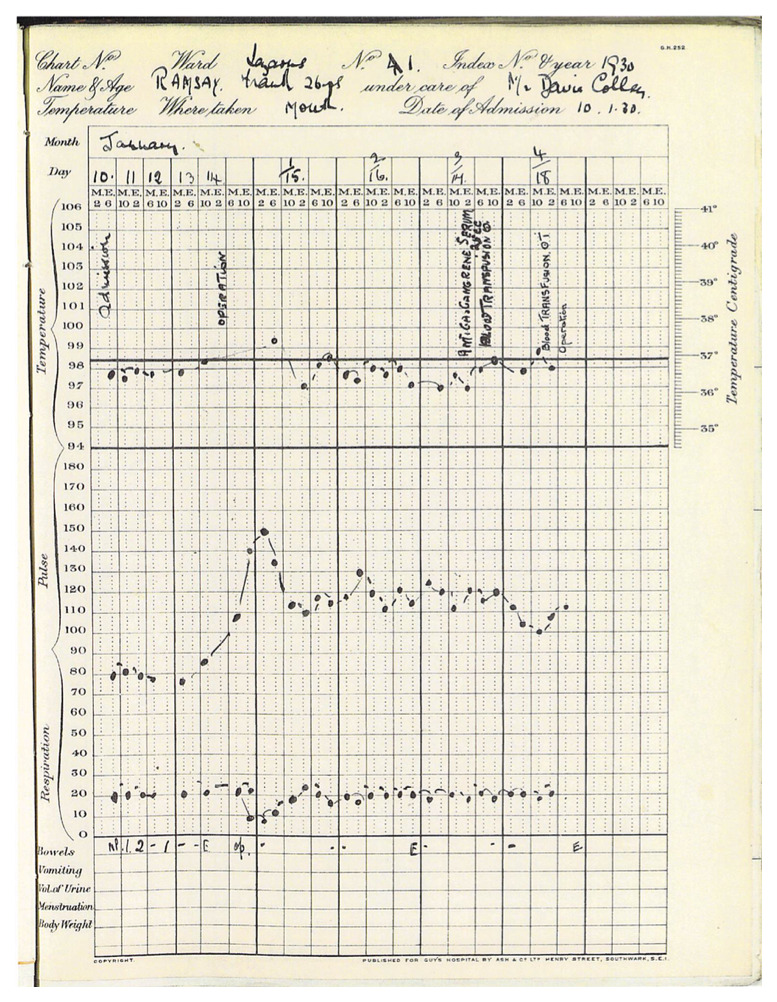
Frank Ramsey’s hospital chart (reproduced with permission from surviving relatives) and Caldicott Guardian at Guy's and St Thomas's NHS Trust.

The Notes tell us that upon admission to Guy’s, Frank Ramsey was jaundiced, afebrile, with a heart rate of 80 and respiratory rate of 20 per minute ( Figure 1). Blood tests on admission showed a normal white blood cell count and differential. Bleeding time was reported as normal (using the technician’s blood as the control). There was no haemoglobin level reported on admission, and red cell morphology was described as “slight anisocytosis and a few (halo) cells”. Blood culture was negative, and a Wasserman test for syphilis was also negative. His urine (tested the day after admission) contained bile, which confirms the diagnosis of jaundice without adding further diagnostic information. No calculus was noted on a plain radiograph. Since most gallstones are radiolucent, this negative finding did not exclude that diagnosis. Ramsey was taken to the operating theatre on Day 5.

As the chart shows, Ramsey remained afebrile and hemodynamically stable until the laparotomy. There is no operative note available, but in the immediate post-operative period his pulse rose from a steady 80 to between 110 and 130 beats per minute, though he remained afebrile. Haemoglobin was reported at 58% on January 17 and there is reference to a transfusion that afternoon and a second transfusion the day after. Haemoglobin even in the 1920s was commonly expressed as gm per 100 cc (or per dl in current shorthand) after conversion from percentages derived from colorimetric scales. However, normal ranges were well known with colorimetric methods, and it seems likely that this result was derived from Haldane’s popular hemoglobinometer scale ( Haldane, 1901). If so, on that scale 100% was the normal for young healthy men, corresponding to 13.8g/dl and 58% would represent profound anaemia consistent with acute blood loss.

The clinical notes confirm an impression that Ramsey's condition had become unstable that morning. At 10.55 that day, Ramsey got “Pituitrin”, which is a combination of oxytocin and vasopressin—seemingly the best inotrope available at the time and suggesting that his blood pressure might have been falling precipitously. He also received “anti-gas gangrene serum”, perhaps as the nearest thing available at the time to broad-spectrum antibiotic cover. All this suggests concerns about both post-operative blood loss and sepsis. 

At 6 pm on January 18 ^th^, Day 9 of the admission, a bedside procedure was performed with "gas and oxygen" (the gas was presumably nitrous oxide or ether) and “local infiltration” was performed with procaine borate, a local anaesthetic developed in the UK and introduced into practice in the mid-1920s. A jejunostomy was then performed, but we have no further information about the procedure or why it was deemed indicated. The treatment record shows, however, that after the operation, Ramsey received a single dose each of atropine, strychnine and adrenaline. The events of these days, charted telegraphically and dispassionately in Ramsey's clinical record, leave the modern reader with no doubt that Ramsey was in dire straits. Ongoing nursing notes record that his respiratory status worsened. After his bedside operation he was troubled by persistent hiccups (which can occur following abdominal surgery), and had a brief period of delirium preceding his death on Day 10 of the admission.

Our review of these medical records prompted a re-examination of the provisional differential diagnoses on which we had originally speculated. The notation referencing spirochetes initially appeared to support the surmise of leptospirosis. While “chronic spirochaetae” or “spirochaetosis” was generally used to describe yaws or syphilis, by 1915 it was known that a spirochaetae (spiral-shaped bacterium) was the cause of Weil’s disease ( Alston & Brown, 1937: 743). Ramsey could not have had yaws, since this infection is acquired during childhood through skin contact in remote regions of Africa, South Asia, and the Western Pacific islands. The Wasserman test for syphilis, widely believed to be highly accurate, was negative. The specific mention of spirochetes in Ramsey’s record kept leptospirosis (or Weil's disease or Spirochaetosis ictero-haemorrhagica) high on our list of possibilities—but it fell short of proving that this was the cause of death.

Our team now included four doctors, one of whom was a hepatologist, but the clinical picture remained confusing. Two leptospirosis experts, Dr. Albert Ko and Dr. Joseph Vinetz, kindly agreed to review the case. Both discounted the diagnosis of Weil’s disease, usually defined as the triad of jaundice, bleeding and acute renal failure, given the sub-acute course of Ramsey’s illness. Death from Weil’s disease typically occurs within 2–3 weeks after onset of symptoms, while Ramsey had a prolonged course of illness and jaundice over six weeks, and the death is typically caused by acute renal failure, hemorrhage or other severe manifestations (myocarditis, arrhythmias, vasculitis, stoke, etc), of which there was no evidence. As for the notation regarding spirochetes, both Drs. Ko and Vinetz commented that the description of spirochetes on dark-field microscopy at the time often represented a false positive. In sum, contemporary experts in leptospirosis placed little value on the new piece of evidence (“spirochetae”) because the disease course did not fit the overall pattern and time-course they would have expected—an example of how expert clinical knowledge is built by accumulating and refining ‘illness scripts’ (internalised narratives of how a disease unfolds) ( Schmidt & Rikers, 2007).

An alternative approach to the differential diagnosis of Ramsey’s illness starts with the fact that his physicians and surgeons never arrived at a definitive diagnosis, suggesting they could have been dealing an entity with which they were unfamiliar. Neither leptospirosis nor primary sclerosing cholangitis appear to have been prominent in the thoughts of clinicians of the day and there is some evidence that these conditions were sometimes missed.

Diseases caused by Leptospira bacteria, for example, were not very well-recognized in England at the time of Ramsey’s illness and hospitalization at the turn of 1929-30. The organism had been isolated for the first time in that country in 1922; in 1924 there had been an outbreak in East Lothian coal-miners. Alston & Brown (1937) described the epidemiology of Weil’s disease and bemoaned the fact that there were very few papers about it in the 1920s, with interest sparked in the disease only in 1934 when it was shown that infection occurred among sewer workers in London. It was by then known to be carried by rats and their urine, with cases arising also in “fieldwork… fish-cleaning and bathing in fresh water” (1937: 741). Only in 1935 was there an effort to authenticate British cases and to try to estimate the proportion of cases of jaundice that were leptospirosis and how many asymptomatic cases there were. Alston and Brown concluded that “It is probable … that the disease is much more prevalent than our present records testify” and that “infection may produce disease of different degrees of seriousness or no observable disturbance of health.” (1937: 3–4) Diagnosis of any spirochetal infection before the advent of serology was also difficult. It depended on silver stains or appearance under light microscopy—one species of spirochete could not be reliably distinguished from another by that method. The notation of “chronic spirochaetae” in Ramsey’s medical record indicates a microscopic finding, perhaps from urine or from samples taken during the laparotomy, but there is no evidence that his physicians connected this finding to his illness.

Similarly, while primary sclerosing cholangitis appears to have first been described in Germany in the mid-1800’s, the condition featured mostly in scattered case reports and was poorly characterized for the next century ( Eaton *et al.*, 2013). At the time of Ramsey’s illness, surgeons recognized that biliary strictures could develop post-operatively (usually after cholecystectomy) or in association with gallstones, but reports of diffuse generalized involvement of the extrahepatic biliary ducts not associated with surgery or stones were rare. The first well documented case in the English language appeared in 1927 ( Miller, 1927), with only around two dozen additional reports of spontaneously arising extrahepatic strictures being described over the next 40 years ( Warren *et al.*, 1966), and in the pre-electronic era, most clinicians would not have had access to these. Until more sophisticated imaging of the biliary tree became possible in the 1960s and 1970s with adoption of endoscopic retrograde cholangiography, the disease was generally not distinguished from far more common causes of biliary strictures in surgical case series and reviews. Lacking an operative note, we cannot say what the surgeon saw when inspecting Ramsey’s biliary tree, other than the absence of stones. But as with leptospirosis, primary sclerosing cholangitis as a potential diagnosis was very unlikely to be on the differential diagnosis docket for a physician or a surgeon in 1930, and might therefore explain both the decision to proceed with a laparotomy and the lack of clarity then and now about the cause of Ramsey's initial illness and subsequent death.

Indeed, no single diagnosis considered by our group of co-authors seems to fit completely with what is known of Ramsey’s illness trajectory, the findings described by his family and doctors, and the fact of his death. The course was not fulminant enough for Weil’s disease and while the more common and milder form of leptospirosis certainly fit with the initial presentation, this form is typically self-limiting and short-lived. Primary sclerosing cholangitis readily explains painless jaundice in the absence of acute liver failure, but the lack of associated and antecedent symptoms would be atypical and the course of illness generally plays out over years, not weeks. Other causes of acute obstructive jaundice, benign, infectious, and malignant, would fit, but tend to be extremely rare in young people and would also have gone unrecognized by Ramsey’s doctors. Even an unusually prolonged course of hepatitis A could explain Ramsey’s course up until his surgery, but it would not fit with descriptions of his internal organs. Once a diagnosis does not provide a unifying explanation for all of the “facts” of a case, a wide variety of diagnoses can, with varying degrees of plausibility, be made to fit.

One of the most important features of medical diagnosis is how the illness progresses over time. In Ramsey’s case, had his medical team been able to watch the illness play out, it would likely have aided them in making a diagnosis. Leptospirosis would have resolved, as very likely would hepatitis A. Primary sclerosing cholangitis would have grumbled along, perhaps taking years to progress to end-stage liver disease. But as fate would have it, the natural history of Ramsey’s disease was interrupted by a well-intentioned surgeon seeking a remediable cause of persistent jaundice.

## Reflections on the clinical reasoning

The history alone raised some questions about the decision to operate on Ramsey. After viewing the case notes, the chart of Ramsey’s vital signs ( Figure 1), and particularly the events of January 17 ^th^ and 18 ^th^ 1930, it seems even clearer that the surgery was the proximal cause of his demise. If his illness was due to a mild form of leptospirosis or prolonged viral hepatitis, Ramsey’s prognosis without the procedure would have been good. Even primary sclerosing cholangitis and other non-malignant causes of obstruction would have been expected to have a reasonable short-term prognosis without surgical intervention, though the former would have ultimately proved fatal in an era before liver transplantation.

However, it cannot be straightforwardly asserted that the decision (made by a combined medical and surgical team) to operate was an error of judgment. Using only the evidence available to them at the time—a clinical suspicion of gallstones, an unwell and non-improving patient, a negative test for a key competing diagnosis (syphilis), and little else in their armamentarium—Ramsey’s doctors may have reasoned that the *risk* of precipitating a decompensation was more than balanced by the *chance* of finding and rectifying a treatable cause for the illness. Whilst there is little doubt with hindsight that the surgical procedure precipitated Ramsey’s death, it is much harder to judge whether the decision to operate was a reasonable one at the time. 

These days, surgeons in training are taught to weigh up the pros and cons of every operation and discuss these with patients before obtaining written informed consent to proceed. This is partly because a great deal of research since Ramsey’s day has demonstrated that many conditions which used to be routinely treated surgically (for example, recurrent tonsillitis, some kinds of coronary artery occlusion, menorrhagia, and even some presentations of appendicitis) have an equal or better prognosis if managed without a surgical procedure. Although back in 1930, medical and minimally invasive alternatives to major surgery were far fewer and surgeons’ confidence in their ability to improve the patient’s outlook was perhaps somewhat stronger than it is now, the fact that Davies-Colley consulted with a medical colleague to deliberate on whether to operate or not suggest that the pros and cons were being weighed very carefully. Unfortunately, the notes do not offer any delineation of the clinical reasoning for the decision to operate, and we are left to fill in the blanks. 

The challenge faced by Ramsey’s physicians and surgeons is plain: they were faced with a patient with an illness they could not quite explain, could not definitively diagnose, but which appeared unremitting. Under such circumstances, a reasonable clinician would be bound to ask: what could we be missing *that might be reversible?* Two questions are pertinent here—how serious the condition is and how likely it is to be present in this particular case? A potentially reversible cause of death becomes impossible to ignore, even if it remains low on our list of differential diagnoses, and particularly if the trajectory of the patient is perceived to be steadily downhill. In Ramsey’s case, the most salient reversible condition was choledocholithiasis, an impaction of a stone in the common bile duct. Some features of the illness pointed away from this, such as the apparent lack of abdominal pain, but again Ramsey’s illness did not fit well with any available alternative diagnosis.

In an era where additional diagnostic tools, such as ultrasound, endoscopy and contrast-enhanced CT scanning did not exist, no active alternatives to surgery were available. Davies-Colley and his colleagues were likely influenced by a strong psychological compunction to *do something* for their patient rather than manage him expectantly. This bias toward intervention continues to permeate medical and surgical practice ( Foy & Filippone, 2013). To not operate would have required them to watch a previously vital and brilliant young man become increasingly jaundiced and – they may have assumed – eventually die. A decision not to operate, only to later discover a stone on a subsequent post-mortem examination would have weighed heavily on the mind. So given the possibility that a non-calcified stone sat wedged in the common bile duct, they chose to operate.

Another influence on the psychological compunction to do something (i.e. operate) is the extent to which doctors of the day might have underestimated the possible *adverse* effects of surgery. They clearly thought that surgery might improve the patient significantly (a positive impact of surgery), and they must have contemplated the possibility that they may not find a stone (neutral impact of surgery), but perhaps they did not fully factor in the possibility that surgery would have a severely *negative* impact on the patient’s trajectory. Put another way, Ramsey's doctors would not have ignored the risks of operating, but, in the face of uncertain evidence and with the wisdom of retrospection, they misjudged them. One can only imagine the disappointment and resignation, but not surprise, when no stone was found—and how the resignation turned to dismay when the patient’s post-operative course turned stormy. 

## Ramsey on counterfactuals and scientific hypotheses

We have employed counterfactual reasoning in re-examining Ramsey’s cause of death—reasoning of this sort: If the diagnosis had been leptospirosis, viral hepatitis, or some benign causes of obstruction and if the laparotomy had not been performed, then Ramsey would have been expected to survive his illness. Some might think these spurious kinds of post-hoc reasoning. But Ramsey himself gave us a way of understanding the rationality of counterfactual conditionals. He suggested that we can evaluate a conditional ‘if *p had been the case*, then *q would have been the case.*’ What is now known as the Ramsey Test for Conditionals is a method for determining whether we should believe a such conditional. We add *p*, hypothetically, to our given body of belief. If the acceptance of *p* leads to a contradiction within that body of belief, we make adjustments, as minor as possible, in order to restore consistency. Then we ask whether *q* is acceptable in the revised body of belief. On this test, ‘Ramsey would have survived his illness if he hadn’t been operated on’ does indeed seem acceptable.

 There are other philosophical issues with the clinical reasoning in our review that would have interested Ramsey the philosopher. How did the clinical understanding of this patient's illness evolve as his doctors assessed him, took measurements, sent tests, and contemplated what was the most likely diagnosis? How did that partial and evolving understanding influence the key clinical decisions and actions which likely contributed to his untimely death? 

 The link here is to Ramsey's position as a self-declared philosophical pragmatist—a philosopher who starts not with the quest for certainty, but with what he called a “human logic” (1926 (1990): 87). For instance, when he was an undergraduate, he challenged one of his eminent mentors, the great economist J.M Keynes, on the nature of probability and induction. In his *Treatise on Probability* ( 1921) Keynes had argued that there is one true probability holding between any two propositions—an objective relation about a degree of partial entailment, part of the formal machinery of drawing conclusions from premises. The young Ramsey argued that there are no objectively fixed probability relations—propositions do not stand in such logical relations to each other. As he put it, there is no such probability as the probability that ‘my carpet is blue’ given that ‘Napoleon was a great general’ ( Ramsey, 1922: 220). Ramsey argued that reasonableness is a matter of having beliefs or habits that work well. Induction is justified not because it is objectively valid, but because it is an indispensable habit, leading, on the whole, to success when we act.

 Later, in his 1926 paper “Truth and Probability” ( Ramsey, 1926), he offered an account of how to measure partial belief by using a subjective interpretation of probability. Degrees of belief can be measured and assessed by examining the disciplined connection between the inner states of beliefs and desires, on the one hand, and the outer states of behaviour, action, and success, on the other. Beliefs are bets that play out in action in the world, and can be evaluated in terms of their success. This paper became a classic, as it was the first time someone had provided a measure and logic of partial belief, as well as a model of subjective expected utility. Ramsey’s foundational work on partial belief underpins elements of contemporary economics, Bayesian statistics, evidence-based medicine, and much else.

 Ramsey, however, was sceptical about applying this highly idealized model to the real world. For one thing, we have imperfect evidence for our beliefs. For another, we can’t measure degrees of belief with precision. Hence no human can live up to the standard of keeping their degrees of belief consistent with the mathematics governing probability. An ideal agent, having full or certain beliefs about every single thing, might always act in a way that she would expect to maximize utility. But people are far from ideal:

…the ideally best thing is that we should have beliefs of degree 1 in all true propositions and beliefs of degree 0 in all false propositions. But this is too high a standard to expect of mortal men, and we must agree that some degree of doubt or even of error may be humanly speaking justified. (1926 [1990]: 80)

Moreover, human fallibility, in Ramsey’s view, is not a friction that interferes with the smooth working of decision-making, but is the condition of humankind. Human psychology cannot be theorized away, as individuals will make different initial probability assignments, and strength of belief will vary from person to person, in ways partly driven by their psychological traits.

 Ramsey’s notion of human logic, which begins with subjective belief and seeks progressively to refine this belief through experience, experimentation and measurement, reflects with remarkable accuracy how clinical reasoning progresses—both traditionally and in contemporary times. Traditionally, we would begin by evaluating a presenting complaint, usually in the form of a story fragment (‘he went to a feast, and soon afterwards, he developed a fever and then his skin then turned yellow’). We would wonder, ‘what could this be?’—perhaps formulating a preliminary list of differential diagnoses, each of which we believe to be more or less likely (‘prior probabilities’). We would ask questions (‘how did you feel before the feast?’, ‘were you a vigorous child?’, ‘how are your bowels?’), examine the patient (is there a palpable liver edge and is it tender?), and conduct tests from a limited list of options (e.g. plain X-ray, urine for bile), thereby progressively adjusting our subjective beliefs about each differential diagnosis (‘posterior probabilities’). To aid this process, we would draw on our internalised scripts of illness trajectories (the sum total of all the stories of patients with similar or contrasting conditions we had ever managed or seen managed or heard about from colleagues), and applied maxims—that is, shared rules of thumb—which encode risky scripts, such as ‘never let the sun set on a blocked common bile duct’ (because, presumably, many such patients died overnight). Maxims are situationally-specific, and can often be contrasted with counter-maxims that apply in subtly different circumstances—for example, “people who do not need operations rarely improve after having them”. Clinical wisdom is more about selecting *which* maxim to follow than about rigidly adhering to a protocol or guideline ( Hunter, 1996).

 In contemporary times, broadly the same process is followed—with three key differences. First, we are taught to rely less on our subjective assessment (e.g. the liver edge we do or don’t feel meeting our palpating fingers as the patient breathes in) and more on ‘objective’ information such as imaging and laboratory tests, which are both more numerous and more accurate than in Ramsey’s time (witness the battery of modern invasive imaging tests today’s clinicians would have offered Ramsey). Second, we are now encouraged to put numbers to our prior and posterior probabilities for a particular patient by using generalised probabilities generated in large randomised controlled trials and observational studies of patients judged to be compatible to our own (a patient in his 20s with deteriorating jaundice following a fever has an x% chance of Weil’s disease and a y% chance of primary sclerosing cholangitis; ( Jaeschke *et al.*, 1994)) and to synthesise such data into clinical decision rules for guiding next steps ( McGinn *et al.*, 2000). Third, we are encouraged to quantify by how much a particular positive or negative test should increase or decrease our subjective belief in a particular diagnosis—the so-called *likelihood ratio*. Indeed, Ramsey would have approved of the contemporary fashion of drawing receiver operator characteristic (ROC) curves of likelihood ratios depending on the chosen cut-off value for distinguishing disease from health in continuously varying parameters ( Søreide *et al.*, 2011). He would not have been surprised that very few tests definitely rule in or rule out a diagnosis—they just make that diagnosis more or less likely depending on the observed result.

 Ramsey would, perhaps, also have something to say about how much we should resist or succumb to our subjective hunches in this brave new world of high-tech imaging, clinical decision rules, likelihood ratios and ROC curves. The empirical Bayesian pretensions of many of today’s clinicians often consist of more or less generalizable probabilities from the relevant literature (true for many images, lab tests, and RCT results) combined with subjective probabilities and partial beliefs gleaned from our own experience and interpretations of the history and physical findings. The decision-making process remains a potpourri of deductions, inductions, and abductions that gels into a clinical Gestalt. ^1^ And trying to explain Gestalt A versus Gestalt B is like trying to trace the outlines of one of those maddening duck-rabbit shadow-grams that Ramsey’s friend Wittgenstein made famous – not least because, as all kinds of literature shows, the Gestalt is shaped by context and values (with specialty training and practice setting as factors that alter both).

 In the year of his death, Ramsey wrote a flurry of papers that started to carve out a view of scientific laws, theoretical terms, and causal statements that went against the prevailing objectivist winds. We should not “take the propositions we make in science and everyday life, and try to exhibit them in a logical system with primitive terms and definitions” ( Ramsey, 1929a: 7). Here, he was referring to what the logical positivists (inspired by Wittgenstein) were doing in 1929: trying to build all knowledge on a foundation of certainty. In contrast, Ramsey argued that we have to see “the vagueness of the whole idea of understanding, the reference it involves to a multitude of performances any of which may fail and require to be restored” ( Ramsey, 1929a: 2). We “are forced to look not only at the objects which we are talking about, but at our own mental states. … we cannot neglect the epistemic or subjective side” (1929a: 6-7). Scientific and causal laws are habits or rules with which we meet the future ( Ramsey, 1929b: 1990: 149). With all the generalised probabilities available to us in the entire medical literature, we five co-authors still needed to consult experts in leptospirosis to make sense of what the history and test results *meant* in Ramsey’s case. All the clinical decision rules in the world cannot substitute for the *subjective* component of clinical expertise and wisdom. It is also generally true that physicians should not play their hunches as a substitute for following the algorithm. Hence the delicate balancing act that characterises (or should characterise) evidence-based-medicine.

 For their part, Ramsey’s medical team struggled to find a unifying Gestalt for the disparate information in their possession. They clearly thought syphilis a possibility, perhaps because of its prevalence, perhaps because Davies-Colley knew his niece and her husband were part of Bloomsbury’s swinging sexual scene. They ruled that out with the Wasserman test and turned to alternatives they could act on—with surgical procedures. Those procedures ruled out the further hypothesis of the blockage. When the time came to list official causes of death, it appears the case was forced to fit within the taxonomy of disease available to them at the time—an early version of the International Classification of Diseases ( Bowker, 1996), even though Ramsey's clinicians were unable to definitively give a name to what it was that ailed him.

 Ramsey would likely have cautioned us regarding our initial quest to diagnose his pre-terminal illness, and would not have been at all surprised by its indeterminate outcome. It is unlikely that we or our successors will arrive at a definitive truth about the question: *what was the illness that led to Ramsey’s death?* But such a definitive truth was something about which Ramsey gave us reasons to be suspicious. Our conclusion will always be underdetermined by the data, and the data we have will almost always accommodate more than one theory. A conclusion will be probabilistic and the only way we can evaluate it is in terms of its being a rule that meets the future well. Ramsey’s medical team met the course of his disease progress with rules that failed them and their patient. But they, like us, were making decisions on the basis of partial belief, the available evidence, and the current theories. From beyond the grave, Ramsey tells us to not be in thrall to the current theory, to understand that our degrees of belief are only as good as the evidence (both subjective and objective) that informs them, and that our decisions are freighted with psychology and our ongoing theories.

 The quest for certainty in clinical medicine, Ramsey tells us, is a fool’s errand. Patients and clinicians alike would do well to remember the uncertainty that must permeate medicine. And clinicians, in particular, would do well to improve upon those aspects of clinical reasoning that incorporate probabilities and belief, rather than searching for definitive algorithms or defaulting to rules that are poorly applicable to the case at hand.
