# Supplementary material for: Case Report: What—or who—killed Frank Ramsey? Some reflections on cause of death and the nature of medical reasoning
Source: Wellcome Open Res. 2023 Nov 20;7:158. Originally published 2022 May 23. [Version 2] doi: 10.12688/wellcomeopenres.17759.2 (PMC10369008; doi:10.12688/wellcomeopenres.17759.2)
Supplement: Peer review history

# Reviewer report: Reviewer response for version 1

**DOI:** 10.21956/wellcomeopenres.19656.r63442

**Published:** 2023-08-07

**Authors:** Kathryn Jack

**Affiliations:** 1 National Institute for Health Research Nottingham Biomedical Research Centre, Nottingham University Hospitals NHS Trust, Nottingham, UK

I’d like to thank the authors and editors for this opportunity to review such an intriguing case-study that is woven into Frank Ramsey’s, (the patient), philosophical teachings. However, more importantly I’d like to thank Stephen Burch for permitting this public scrutiny of his grandfather’s illness and death. All case studies, however old, give clinicians the opportunity to reflect, learn, and consider how their own practice (be that clinical, education, research, or any combination) could be improved. I’m not a philosopher, but an academic nurse, so my review is focused through this lens. 

 In keeping with the style of reviewer number one, my review of this fascinating story is a short commentary. The manuscript meets all of the usual domains required for a case study with one possible exception, that being the limited clinical details reported. However, I am highly mindful of the need to protect Frank’s confidentiality, and of the likely scant medical and nursing records that were both recorded in writing at that time and retained in medical notes. Therefore, I consulted historical textbooks (Ashdown, 1929; Morten and Taylor, 1942) to further illuminate the nursing and medical thinking at that time. 

 According to the available records, Frank was admitted to hospital with a prolonged episode of painless jaundice. The textbooks explain that jaundice is always obstructive, either due to biliary duct blockage from gallstones or compression of smaller bile ducts due to tumours, cirrhosis, or catarrh [inflammation] (Ashdown, 1929). Cirrhosis is caused by alcohol and occurs in people aged over 40 years (Ashdown, 1929), and catarrhal jaundice is the most common cause in younger people (Morten and Taylor, 1942). The hospital chart shows that blood pressure measurement was not a regular clinical observation at that time which further blurs the clinical picture for contemporary interpretation. Nurses at that time however would have noted the quality of the palpated pulse with “bounding” and “thready” being an unscientific and often inaccurate way of indicating high or low blood pressure respectively. Nursing care, then and now, places high emphasis on the importance of maintaining asepsis and cleanliness, but sterility standards were not as they are today. For example, in addition to the initial laparotomy it is noted that Frank received two blood transfusions, and these would have been undertaken thus: “both blood donor and recipients’ skin incised, vein incised, glass cannula inserted into veins, 20ccs blood drawn from donor in a syringe and injected into patient, continue as necessary” (Ashdown, 1929, p.70). Furthermore, the bedside jejunostomy (opening an area of small bowel), however well intentioned, would not have met today’s standards of an aseptic procedure. The overall risk of Frank developing sepsis during this hospital admission is high and whilst he was apyrexial, I wonder if the more recently described phenomenon of “cold” sepsis may also have contributed to the outcome (Rumbus, 2017). 

 A number of infectious and hepatic causes of jaundice have been discussed and discounted. However, I am drawn back to the letter from Lettice reporting that not just the liver, but kidneys too, were in a “frightful condition”. A case study by Zang et al (2020) report the story of a man with polycystic liver and kidney disease (a rare genetic condition). The resultant obstructive jaundice could be due to the cysts squeezing the bile ducts, but alternatively could be due to the bile duct malformation that can occur in this condition. Any intra-hepatic irregularities may not be easily visualised via a laparotomy and there is no postmortem histopathology report. 

 The exact cause of Frank’s jaundice may never be known. However, it is easy to agree with the authors that the surgical interventions hastened his death. 

 Turning to the philosophical section of this paper, Frank’s explanations about partial beliefs and probability are very clearly aligned with contemporary health care decision making. The saying (appropriated from opera) that “ it’s not over until the histopathologist has sung” certainly follows the maxim that subjective belief is refined through experience, experimentation, and measurement. Additionally, the starting point of subjectivity and progressive refinement is reminiscent of the retroductive form of reasoning proposed by Bhaskar (2016, p.3), this being a disciplined imaginative process whereby the researcher develops the causes or unseen generative mechanisms which if existed would explain the outcomes observed. 

 There is a growing appetite amongst healthcare researchers, particularly nurses and other non-medical allied healthcare professionals,  to conduct mixed methods research underpinned by pragmatism.  Frank’s philosophical position can certainly contribute to academic discussions in this field. With regards to what could be learnt from this case study for nurses, apart from the importance of documentation, the need to deliver care grounded in evidence remains paramount and our professions’ contemporary role as patients’ advocate supports this action. 

 Ashdown, AM. (1929). A Complete System of Nursing. 9th Ed.  JM Dent and Sons: London

 Bhaskar, R. (2016). Enlightened Common Sense. The Philosophy of Critical Realism. Oxon: Routledge.

 Morton, H and Taylor, F. (1942). The Nurse’s Dictionary. 19th Ed. Faber and Faber Ltd: London

 Rumbus Z, Matics R, Hegyi P, et al. (2017). Fever Is Associated with Reduced, Hypothermia with Increased Mortality in Septic Patients: A Meta-Analysis of Clinical Trials. Plos one. 12(1):e0170152. DOI: 10.1371/journal.pone.0170152. 

 Zhang L, Gan L, Liu Q, Li Y, Lin J, Ou S. (2020). Obstructive jaundice in a patient with polycystic liver disease complicated with polycystic kidney and polycystic lung: A case report. Medicine (Baltimore). 99(14):e19511. doi: 10.1097/MD.0000000000019511.

Are enough details provided of any physical examination and diagnostic tests, treatment given and outcomes?

Partly

Is the case presented with sufficient detail to be useful for other practitioners?

Yes

Is sufficient discussion included of the importance of the findings and their relevance to future understanding of disease processes, diagnosis or treatment?

Yes

Is the background of the case’s history and progression described in sufficient detail?

Partly

Reviewer Expertise:

Liver disease, health inequalities, mixed methods research

I confirm that I have read this submission and believe that I have an appropriate level of expertise to confirm that it is of an acceptable scientific standard.

## References

[1] : A Complete System of Nursing. 9th Ed. *JM Dent and Sons: London* .1929;

[2] : Enlightened Common Sense. The Philosophy of Critical Realism. *Oxon: Routledge.* .2016;

[3] : The Nurse’s Dictionary. 19th Ed. *Faber and Faber Ltd: London* .1942;

[4] : Fever Is Associated with Reduced, Hypothermia with Increased Mortality in Septic Patients: A Meta-Analysis of Clinical Trials. *PLoS One* .2017;12(1) : 10.1371/journal.pone.0170152 e0170152 10.1371/journal.pone.0170152 28081244PMC5230786

[5] : Obstructive jaundice in a patient with polycystic liver disease complicated with polycystic kidney and polycystic lung: A case report. *Medicine (Baltimore)* .2020;99(14) : 10.1097/MD.0000000000019511 e19511 10.1097/MD.0000000000019511 32243367PMC7220720

# Reviewer report: Reviewer response for version 1

**DOI:** 10.21956/wellcomeopenres.19656.r63446

**Published:** 2023-08-01

**Authors:** John Capps

**Affiliations:** 1 Department of Philosophy, Rochester Institute of Technology, Rochester, New York, USA

This account of Frank Ramsey’s last days is a thought-provoking combination of history, medicine, philosophy, tragedy and mystery. My comments have little to do with substance—the article is clearly written, carefully researched and argued, and peels back the layers of a sad episode in 20th century intellectual history—and more to do with some of the thoughts it provokes. While Ramsey might not be thought of primarily as a philosopher, his death is another instance of philosophers meeting untimely and tragic ends. This all started with Socrates (hemlock), continued through Descartes (supposedly the victim of a pre-dawn teaching schedule), and happened more recently to Moritz Schlick (murdered by a student whom the Nazis later released from prison) and Janina Hosiasson-Lindenbaum (arrested by the Gestapo and executed in 1942). The list goes on.

     As Cheryl Misak describes in her 2020 biography *Frank Ramsey: A Sheer Excess of Powers*, Ramsey’s ideas had and continue to have an impact across a variety of fields: mathematics, economics, philosophy, and computer science, to name a few. Perhaps he was lucky or just very good, but he seemed to have had a knack for being in the right place at the right time, rubbing shoulders and carrying on conversations with influential intellectuals and public figures of the post-WWI era. Or: as this article shows, he was lucky until he wasn’t, dying at—by our contemporary standards at least—the shockingly young age of 26.

     Ramsey was super-smart but his early death is surely part of his mystique. There’s a sense of doom and tragedy that hangs over his story. It’s refreshing that the authors approach his death not merely as a depressing piece of trivia but as an opportunity to draw connections to Ramsey’s own views about decision-making under uncertain conditions. That’s where the story also becomes a mystery: as their title puts it, “what—or who—killed Frank Ramsey?” Was it Leptospirosis? Primary sclerosing cholangitis? Or was it a well-intentioned but ultimately fatal surgical intervention?

     We might also wonder why and whether we should care about the cause of Ramsey’s death. I don’t mean this to sound flippant: I think there are some interesting philosophical questions in the vicinity that are worth considering. For one thing, the authors note that “it is unlikely that we or our successors will arrive at a definitive truth about the question: *what was the illness that led to Ramsey’s death*? But such a definitive truth was something about which Ramsey gave us reasons to be suspicious.” I’m not sure if this means that definitive truths are suspect *in general* or merely that, in this particular case, the definitive truth is out of reach. I’m also not sure whether to put the stress on *definitive* or *truth*. If the former this would suggest that we can arrive at the truth but without the certainty to call it “definitive”; if the latter, this would suggest that there is nothing that we could call the “truth” about Ramsey’s illness.

     I take the authors’ point to be that there are factors in this *particular* case that put the definitive truth out of reach: most likely a combination of historical distance, missing paperwork, and the uncertainty that, as they point out, “must permeate medicine.” Moreover, I don’t think they are denying that there is a truth about Ramsey’s illness. Rather, Ramsey’s illness seems to be an example of what C.S. Peirce (one of Ramsey’s influences) called a “buried secret.” “Buried secrets” pose a problem for Peirce (and maybe for Ramsey too) because he defined truth in terms of what would stand up to unlimited levels of scrutiny. For example, to say that it is true that Ramsey was born in 1903 is just a more efficient way of saying that this claim would be supported by the evidence no matter how long or how hard one looked (by consulting Wikipedia, finding his original birth certificate, digging up obituaries, reading the biography…). The problem of buried secrets, then, is that if a claim resists scrutiny—perhaps because it is about the distant past or about trivial matters that no one bothered to record—then on Peirce’s account it would seem to be neither true nor false. That will strike a lot of people as an obvious mistake because we tend to think of truth and knowability as different properties: a claim can be unknowable but still be true. Peirce’s own response to this problem wasn’t always clear but he did claim that “followers of science are animated by a cheerful hope” that the truth can always be unearthed and Ramsey seemed to agree with this, at least with respect to the types of counterfactuals discussed in this article. While we can’t rule out the possibility of buried secrets, “followers of science” are bound to assume that there’s no secret that cannot be revealed given enough time, energy, and ingenuity. This would apply to the Ramsey’s final illness as well: even if the *definitive* truth is out of reach we hope there’s still a truth to be discovered.

     So, why do we (or some of us) care to discover the truth about Ramsey’s illness and death? We don’t care for any of the typical and familiar reasons: Ramsey isn’t a personal friend or relative, there is no living person to be held responsible or punished, and understanding his cause of death won’t have any direct effect on current clinical practice. Lots of people— *lots* of people—die too soon, and under much worse circumstances. And, yet, we care about Ramsey.

     The reason we care about Ramsey and his illness is connected to why we care about the truth. If we’re pragmatists like Ramsey then we don’t care about the truth merely for its own sake. Fundamentally, we care about the truth because we care about getting things right, and we care about getting things right because we want to achieve our goals. One goal of medical professionals is to uphold the Hippocratic Oath: do no harm. The authors of this article argue convincingly that Ramsey died because a surgeon just couldn’t help himself. He felt compelled to operate and did harm as a result. Perhaps Ramsey’s medical team did the best they could. That’s what makes this a tragedy.

     Hope plays a role here as well. We hope that we can do better than those who came before. We hope that when we get things right we *really* get them right. But what *reason* is there for this hope? Why prefer hope over despair? It’s here that Ramsey himself has something to add:

- I find, just now at least, the world a pleasant and exciting place. You may find it depressing; I am sorry for you, and you despise me. But I have reason and you have none; you would only have a reason for despising me if your feeling corresponded to the fact in a way mine didn’t. But neither can correspond to the fact. The fact is not in itself good or bad; it is just that it thrills me but depresses you. On the other hand, I pity you with reason, because it is pleasanter to be thrilled than to be depressed, and not merely pleasanter but better for all one’s activities. (1990: 249-250)

Ramsey’s point is that hope and optimism are not only “pleasanter” than the alternatives but that they make us more effective agents (“better for all one’s activities”). It’s this hope that animates the search for truth, for getting things right, and for doing better. I suspect it’s also what motivates this article as well.

Are enough details provided of any physical examination and diagnostic tests, treatment given and outcomes?

Yes

Is the case presented with sufficient detail to be useful for other practitioners?

Yes

Is sufficient discussion included of the importance of the findings and their relevance to future understanding of disease processes, diagnosis or treatment?

Yes

Is the background of the case’s history and progression described in sufficient detail?

Yes

Reviewer Expertise:

History of Philosophy

I confirm that I have read this submission and believe that I have an appropriate level of expertise to confirm that it is of an acceptable scientific standard.

## References

[1] : Epilogue. In: Philosophical Papers, Ed. D. H. Mellor. *Cambridge: Cambridge University Press* .1990;245-250

# Reviewer report: Reviewer response for version 1

**DOI:** 10.21956/wellcomeopenres.19656.r63444

**Published:** 2023-07-25

**Authors:** Mathew Mercuri

**Affiliations:** 1 Department of Medicine, McMaster University, Hamilton, Ontario, Canada

The authors explore an interesting case of the death of Frank Ramsey to highlight the very lessons to be learned in his own philosophy that bear on clinical reasoning and decision making. One goal of the manuscript is to settle on what or who was the cause of Ramsey’s death. The answer provided by the authors is not definitive, although the data seems to converge on the surgery he underwent as the culprit. The manuscript provides excellent detail about the authors’ causal reasoning and is well researched. I very much appreciate the effort to not only examine Ramsey’s medical file, but to also bring in and deliberate with experts about diseases that serve as potential diagnoses so to cover as broad a perspective as possible. I have no concerns about the quality of the analysis and think this manuscript is very well done in all respects. I have only a few comments that I believe are worth some additional attention in thinking about this case, none of which should prompt changes to the manuscript as written.

 An interesting question not explored in the manuscript is that of responsibly – the clinicians were likely in error, but is that error reasonable? I would argue yes – even with the knowledge of today and a wide network of experts, the authors illustrate how much judgment plays a role in ruling out each of what come across to this reader (and seemingly to the authors and their colleagues) as reasonable alternatives. Judgment by clinicians seems unavoidable in clinical decision making. The authors imply enthusiasm about the benefits of surgery could have played a role in the judgment of Ramsey's physicians and surgeon and that perhaps “they did not fully factor in the possibility that surgery would have a severely negative impact on the patient’s trajectory”, or rather, they misjudged the risks. This point raises a reasonable explanation for how Ramsey could end up in surgery, but it is conjecture. For example, it is possible that his surgeon had a good estimate of the risk and appreciated the possibility of there being no stone present, but still felt that on the balance of probabilities (or something akin to that in the surgeon’s belief based on what was known at the time prior to surgery and the clinical narrative he and the other clinicians established), surgery was the best way forward. It may be that many surgeons of the day (or even today) put in his shoes would have settled on the same decision. If so, would it be fair to say the surgeon is responsible for Ramsey’s death (which is different than saying the surgery is the cause of his death)? I raise this issue mindful that cases that are subject to clinical malpractice suits may share features with the one presented in the manuscript. What could be learned from the analysis of the Ramsey case (and his philosophy) that is relevant to how we deal with malpractice suits in courts of law?

 The second issue I would like to explore is the tension between the “subjective component of clinical expertise and wisdom” and the “algorithm” or actuarial reasoning from formally collected data. The authors state that “all the clinical decision rules in the world cannot substitute for the subjective component of clinical expertise and wisdom”, but also state that “physicians should not play their hunches as a substitute for following the algorithm”. These are two extremes, and it is fear of following bad hunches that to me seems to be a driving factor in and the appeal (to many) of evidence based medicine thinking. What does the Ramsey case tell us about the balance between clinical hunches (e.g, which might be used to build an initial set of differential diagnoses) and clinical decision rules? Is there anything in this case or his philosophy that leads to understanding what is an appropriate balance? Were the clinicians in the Ramsey case playing their hunches or were they making reasonable and calculated decisions based on what were their algorithms for practice? Was it their algorithms that let them down or was it that they did not pay enough attention to them? What could they have done better?

 Overall, I very much enjoyed reading the manuscript and applaud the authors for taking on this case. I feel that the study highlights an important activity in clinical practice, i.e., reflection on practice. That reflection need not be about one’s own practice, as something can be learned from a review of any clinical case, both challenging and seemingly mundane. I am encouraged that such review and reflection is done to some extent in many clinical environments, in particular academic hospitals, in various forms, such as weekly clinical or academic rounds, safety committees for clinical errors, near misses, or sentinel events, etc. I am even more encouraged that the clinician authors engaged in a deep dive of a historical case. Thus, this case is not a purely academic exercise or should not only be of interest to philosophers and historians. It provides real analysis of clinical practice and raises important considerations about reasoning that is highly relevant for both clinical learners and practicing clinicians.

Are enough details provided of any physical examination and diagnostic tests, treatment given and outcomes?

Yes

Is the case presented with sufficient detail to be useful for other practitioners?

Yes

Is sufficient discussion included of the importance of the findings and their relevance to future understanding of disease processes, diagnosis or treatment?

Yes

Is the background of the case’s history and progression described in sufficient detail?

Yes

Reviewer Expertise:

Health Services Research/Epidemiology, History and Philosophy of Science

I confirm that I have read this submission and believe that I have an appropriate level of expertise to confirm that it is of an acceptable scientific standard.

# Reviewer report: Reviewer response for version 1

**DOI:** 10.21956/wellcomeopenres.19656.r50714

**Published:** 2022-07-12

**Authors:** John Gabbay

**Affiliations:** 1 Wessex Institute, University of Southampton, Southampton, UK

Were this to be just a standard referee's report I would simply say in summary that it's a clear and (to me) extremely interesting piece, based on sound methods that are well executed, and it should be published (albeit in a journal with the right audience, which this may not be, but that is another matter: it’s here now, thanks to F1000 publishing).

 My report is not so much a peer review as a commentary, and a rather self-indulgent one at that. I want to engage with some of the authors’ arguments and premises in ways that are not critiques of the paper itself. They are just thoughts that it stimulated (debatable thoughts maybe, but isn't that the point of philosophy?), which the authors and readers might like to think about and perhaps take account of, even though they relate to what anyone familiar with my own work will recognise as Hobby Horses. (Does that count as a minor conflict of interest?)

 I have to confess to my own priors as I began to read the article. Having many years ago been a (Wellcome-funded) medical historian, I groaned inwardly at the prospect of reading an historical diagnosis.  When one maintains, as I did back then and still do now (Hobby Horse #1), that our concepts of disease are social constructs, and that all our observations are theory laden, then surely the idea of retrospectively using our modern constructs to interpret past observations is doomed. We have no choice but to work with the findings of doctors from a different world, who had different theoretical lenses, different norms of practice, different available diagnostic resources and different treatment choices. They were answering questions that literally only made sense in their own context. All of that makes it impossible to achieve a simple “real truth out there” from some objective perspective – to find out "what really happened". I long ago argued that if we want to know, for example, what asthma was like in the C18 ^th^, e.g. whether it had the same characteristics as now, then however hard we try (and I did), we find ourselves unable to do so. (A form of what I labelled “historical paralysis” (Gabbay 1982)).   And even when we think we have indeed reconstructed some past disease, we have almost certainly done so by applying diagnostic criteria based on our modern understanding of patho-physiology, for example, to give that past-perceived/ long-ago-constructed reality a (modern) name, a modern construction – solving a problem that is now re-framed in modern terms. In doing so we have inevitably distorted what happened. Hence I usually ask “What on earth is the point of doing that? Is it telling us something about the past or about ourselves?” All of which explains why I thought there would be precious little point in reading a post-mortem inquiry into a death caused so long ago.  (Moreover – and this is a separate concern – how would we know that we, the clever modern diagnosticians with all our modern knowledge, have finally got the answer right?  Who is to say that in 100 years’ time, when *we* are all history, someone won’t be writing a paper savouring the irony that the authors of this article hadn’t recognised a clear case of Foster-Vinetz syndrome, first described back in 2027?)

 However, these authors have shown me that there can indeed be a point to such an exercise. On two levels. First, reading the early sections was ... fun! It really got my diagnostic juices going (they’d dried up about 40 years ago when I left clinical medicine behind). The exposition of the differential diagnoses and their pros and cons is a great read and I imagine that practising clinicians who read these pages would learn much from it. Second, in the later sections of the paper, this case-history is indeed being explicitly used to solve a relevant C21 ^st^ problem – not a long-forgotten clinical one, but a current philosophical one. The authors use their findings in a very intelligent and illuminating way by analysing the thinking underpinning the medical decisions and actions they describe.  Even just to do that would have been an ingenious stroke, but to do it using the philosophical framework developed by the unfortunate patient? That's terrific! Probably unique (but then, so was Frank Ramsay). 

 I don’t feel qualified to comment on the exposition of Ramsay’s philosophy, but the way the authors approach the clinical-reasoning side of the story is a great read. However, (and here comes Hobby Horse #2), I think it is also largely a misleading fiction. The hypothetico-deductive line of enquiry, the objective solution of a logical puzzle, complete with careful analysis of probabilities and priors, of likelihood ratios and receiver-operator curves…  Really?  In an overstretched hospital or an overworked GP’s surgery? No doctor would survive more than a day behaving that way – and more to the point, neither would many of the patients she never got round to seeing because she was being so rigorously systematic about the few patients that she did ponder at such length. If you want to understand how doctors really think, you’ll need to go beyond an analysis such as this.

 At least the authors claim only that doctors these days are “taught” and “encouraged” to compute prior and posterior probabilities based on RCTs, to quantify the likelihood ratios, and so on. Perhaps they covertly recognise that it’s not often that practitioners actually do all that stuff. What’s described in this paper is the idealised, rationalised version of how doctors think – or should think – not how they normally *do* think. Even if they wanted to, or remembered how to, most clinicians aren’t in any position to indulge in the luxury of such elaborately logical thinking. Nor can they get by (for long!) relying as suggested in the paper on shortcuts and heuristics, on maxims and rules of thumb – which can be error strewn. Nor is their thinking just a Gestalt that (magically?) emerges, as the authors describe it, from a “potpourri”. What clinicians do in practice is both more complicated and more simple than that.  In reality, practitioners’ thinking embodies aspects of all the above – and a lot more besides.  From what we have observed in a series of ethnographic studies, practitioners make their decisions thanks to their clinical mindlines. 

 Clinical mindlines, are “internalised, collectively reinforced and often tacit guidelines that are informed by clinicians’ training, by their own and each other’s experience, by their interactions with their role sets, by their reading, by the way they have learnt to handle the conflicting demands, by their understanding of local circumstances and systems, and by a host of other sources” (Gabbay and le May 2011, page 44). They are not lengthy logical thought processes, nor lazy shortcuts, nor deft ‘intuition’, but a deeply developed embodiment of many types of accumulated knowledge-in-practice-in-context that enables good practitioners to make astonishingly fast, efficient, and flexible decisions.

 Moreover, as our ethnographies showed, a crucial part of the mindlines model is the way in which clinicians consult colleagues whose opinions they trust (Gabbay and le May 2016). Sometimes they do that in addition to going through the literature, sometimes instead of doing so. It’s clear that even the authors of this article didn’t (just) scour everything worthwhile that’s been written about sclerosing cholangitis or leptospirosis or whatever. They talked or corresponded about the problem as a “team”, doubtless eliciting and checking out each other’s assessments, judgements, and experience; they consulted colleagues like Albert Ko and Joseph Vinetz. And that is exactly what most clinicians do, for mindlines are also a collective phenomenon. (In all probability, Colley and his colleagues would have acted similarly in the doctors’ dining room, on the ward round, in theatre, in the library, and striding together down the long hospital corridors.)  

 Andrée le May and I would argue that clinical mindlines are a highly sophisticated and hard-won way of making rapid decisions while taking account of a myriad different kinds of knowledge accumulated over a lifetime of (social, interactive) knowledge-gathering and experience – several lifetimes if you include the colleagues who contribute their experience and expertise to an individual’s mindlines. Mindlines are a kind of collective professional capital that should not be ignored in favour of unrealistic exhortations to rigorous, time-consuming, formal assessment of probabilistic, trials-based information that never quite fits the kind of individual clinical problem that Ramsay posed.  The hyper-rational thinking described in this article is occasionally necessary. But is almost certainly never used alone to manage a condition or patient such as this. Let’s not pretend it is, and let’s celebrate (and try to understand better, and hence improve) the mindlines-mediated, socially-constructed thinking that really shapes most clinical decisions.

 The irony is that a decade ago now, in her endorsement on the cover of the book in which Andrée le May and I elaborated the concept of clinical mindlines, Trisha Greenhalgh was kind enough to call it “one of the most important publications about clinical practice and evidence-based medicine to appear in the last 20 years”.   She and her present co-authors may, therefore, wish to consider at least a nod in the direction of mindlines as they analyse the decision-making processes in such a challenging case as Ramsay’s. 

 Well, I promised a self-indulgent commentary, and I’m sorry for that. But I hope that by trotting out my two main Hobby Horses I may have encouraged the authors and readers of this absorbing article to take the opportunity for a bit of a reality check.

Are enough details provided of any physical examination and diagnostic tests, treatment given and outcomes?

Yes

Is the case presented with sufficient detail to be useful for other practitioners?

Partly

Is sufficient discussion included of the importance of the findings and their relevance to future understanding of disease processes, diagnosis or treatment?

Yes

Is the background of the case’s history and progression described in sufficient detail?

Yes

Reviewer Expertise:

Knowledge mobilisation

I confirm that I have read this submission and believe that I have an appropriate level of expertise to confirm that it is of an acceptable scientific standard.

## References

[1] Asthma attacked? Tactics for the reconstruction of a disease concept, In: P. Wright.

[2] : Practice-based Evidence for Healthcare.2010; 10.4324/9780203839973 10.4324/9780203839973

[3] : Mindlines: making sense of evidence in practice. *British Journal of General Practice* .2016;66(649) : 10.3399/bjgp16X686221 402-403 10.3399/bjgp16X686221 PMC497994927481961
